# SNP rs7430456 mediated LINC00578 drives triple negative breast cancer through regulating miR-143-5p expression

**DOI:** 10.1186/s41065-026-00705-7

**Published:** 2026-07-17

**Authors:** Jing Zhai, Wei Wu, Xiaoyun Mao, Lianjun Wu

**Affiliations:** 1 Radiation Oncology Department, Tianjin Medical University General Hospital, Tianjin City, 300052 China; 2 Department of Breast and Thyroid Surgery, Dazhou Central Hospital, Dazhou City, 635000 China; 3 Department of Breast and Thyroid Surgery, Ganzhou Hospital-Nanfang Hospital, Southern Medical University (Ganzhou People’s Hospital), No.16, Meiguan Avenue, Zhanggong District, Ganzhou City, 341000 Jiangxi Province China; 4 Department of Surgery, The Third People’s Hospital of Cangnan County, No.188, Wenxin Road, Qianku Town, Cangnan County, Wenzhou City, 325804 Zhejiang Province China

**Keywords:** rs7430456, SNP, TNBC, Progression, lncRNA

## Abstract

**Background:**

A large number of cancer-related single-nucleotide polymorphisms (SNPs) are distributed in the genomic regions of long non-coding RNAs (lncRNAs), yet the mechanisms linking them to cancer risk have not been fully clarified so far. This study explored LINC00578 rs7430456’s association with breast cancer susceptibility and LINC00578’s role/mechanism in triple-negative breast cancer (TNBC).

**Methods:**

A total of 480 breast cancer patients and 460 controls were enrolled. LINC00578 rs7430456 genotyping and LINC00578 expression detection via RT-qPCR were performed. Functional assays (proliferation, migration, invasion) were conducted in TNBC cell lines, and the LINC00578-miR-143-5p interaction was explored by dual-luciferase reporter assay.

**Results:**

LINC00578 rs7430456 G allele and AG/GG genotypes reduced breast cancer risk. LINC00578 was upregulated in breast cancer (especially TNBC) with an AUC of 0.873 for diagnosis. LINC00578 knockdown inhibited TNBC cell proliferation, migration, and invasion. LINC00578 sponged miR-143-5p, and miR-143-5p mediated its oncogenic effects.

**Conclusions:**

LINC00578 rs7430456 is associated with breast cancer susceptibility, and LINC00578 promotes TNBC progression via sponging miR-143-5p, being a potential biomarker and therapeutic target for breast cancer, particularly TNBC.

## Background

Breast cancer continues to be the most frequently diagnosed cancer and a principal cause of cancer-related death in women worldwide, with substantial heterogeneity in incidence, prognosis, and therapeutic response across ethnicities and subtypes [1, 2]. Triple-negative breast cancer (TNBC) accounts for ~ 15–20% of all breast cancer cases and exhibits the poorest clinical outcomes due to limited targeted treatment options [3]. Thus, identifying ethnicity-specific molecular markers and unraveling their regulatory mechanisms is critical for improving early diagnosis and personalized therapy of breast cancer, especially TNBC.

Long non-coding RNAs (lncRNAs), defined as RNA transcripts > 200 nucleotides with no protein-coding capacity, play pivotal roles in tumorigenesis and cancer development through modulating gene expression via epigenetic, transcriptional, and post-transcriptional mechanisms [3]. Studies have shown that lncRNAs are related to tumorigenesis [4, 5]. Variations in lncRNA-encoding genomic regions, particularly SNPs, may influence cancer development by disrupting lncRNA expression or function, thereby affecting key oncogenic pathways [6]. Among them, LINC00578 has recently gained attention in breast cancer research: a pioneering study in the Brazilian population identified a single-nucleotide polymorphism (SNP) in LINC00578, rs7430456, as a potential genetic modifier—rs7430456 genotype was associated with both LINC00578 expression levels and breast cancer susceptibility in the Brazilian cohort, suggesting a functional link between genetic variation of LINC00578 and breast cancer risk [7]. However, genetic polymorphisms and their phenotypic effects often exhibit marked ethnic disparities due to differences in population genetics and linkage disequilibrium patterns [8, 9]. Accumulating evidence has demonstrated that such disparities are often driven by pronounced differences in allele frequency distributions across populations. To contextualize the relevance of investigating rs7430456 in Chinese Han individuals, we compared its allele frequencies in our healthy control cohort with publicly available data from the 1000 Genomes Project Phase 3. In the present study, the G allele frequency of rs7430456 in healthy Chinese Han controls was 77.61%, which was highly consistent with the frequency reported for Han Chinese in Beijing (CHB, 78%) and East Asian populations (EAS, 82%) in the 1000 Genomes database. Notably, the G allele frequency in our cohort was substantially higher than in other populations, including African (18%), admixed American (30%), European (39%), and South Asian (59%) populations. This pronounced divergence highlights the unique genetic architecture of rs7430456 in East Asian populations, suggesting that findings derived from other ethnic groups cannot be directly extrapolated to Chinese Han individuals. To date, the expression profile of LINC00578, the association of rs7430456 with LINC00578 expression and breast cancer risk, and its subtype-specific relevance (particularly in TNBC) have not been investigated in the Chinese Han population, the largest ethnic group in China, and a distinct demographic with unique breast cancer epidemiological characteristics.

lncRNAs are increasingly recognized to operate as competing endogenous RNAs (ceRNAs) by binding and inhibiting microRNAs (miRNAs), thereby regulating the expression of downstream target genes [10]. miR-143-5p, a well-characterized tumor suppressor miRNA, is frequently downregulated in breast cancer and inhibits breast cancer cell proliferation, migration, and invasion by targeting oncogenic mRNAs [11, 12]. Furthermore, the molecular mechanisms underlying LINC00578-mediated breast cancer progression remain incompletely understood. Whether LINC00578 exerts its oncogenic effects in breast cancer by sequestering miR-143-5p has not been explored.

To address these gaps, the present study aimed to evaluate the association of LINC00578 rs7430456 genotype with LINC00578 expression and breast cancer risk (with a focus on TNBC) in a Chinese Han cohort. The functional role of LINC00578 in breast cancer cell proliferation, migration, and invasion was investigated, and the potential regulatory mechanism of LINC00578 via targeting miR-143-5p was explored. The findings of this study will provide new insights into ethnicity-specific breast cancer biomarkers and molecular targets for LINC00578-related breast cancer therapy.

## Methods

### Study participants

A total of 480 breast cancer patients and 460 healthy controls were enrolled in this study. All participants were of Chinese Han ethnicity. Breast cancer patients were diagnosed histopathologically, and their molecular subtypes (Luminal, HER2-positive, TNBC) were determined. Sample size was calculated by GPower 3.1.9.7 with post-hoc analysis for power analysis, and the power value for the present sample size was 0.976, and the result greater than 0.8 proved that the sample size was sufficient. Health controls had no history of cancer and were frequency-matched to breast cancer patients by age. Inclusion criteria for breast cancer: pathologically confirmed primary breast cancer. Exclusion criteria: concurrent malignant tumors, history of prior radiotherapy or chemotherapy, or incomplete clinical data. Exclusion criteria for health controls: history of breast disease, history of cancer, or history of breast-related surgery. Clinical data, including age, age at menarche, age at menopause, menopause status, number of pregnancies, number of abortions, breastfeeding history, and family history of cancer, were collected through structured questionnaires. This study was approved by the institutional review board of the Dazhou Central Hospital, and written informed consent was obtained from all participants.

### Genotyping of LINC00578 rs7430456

Genomic DNA was isolated from peripheral blood with a commercial kit (Qiagen, Hilden, Germany). Genotyping of the LINC00578 rs7430456 polymorphism was conducted using the TaqMan SNP genotyping assay (Aaasy ID: C__32476934_10, Applied Biosystems, USA) on the StepOnePlus Real-Time PCR System. The reaction mixture (10 µL) included 5 µL TaqMan Universal PCR Master Mix (2×), 0.5 µL genotyping assay mix (40×), and 10 ng genomic DNA. PCR amplification conditions: 10-minute pre-denaturation at 95 °C; 15 s of denaturation at 95 °C, followed by 1 min of annealing/extension at 60 °C, for a total of 40 cycles. Hardy-Weinberg equilibrium was assessed in controls with a chi-square test.

### RNA extraction and quantitative real-time PCR (RT-qPCR)

Total RNA was extracted from serum samples and cell lines using TRIzol reagent (Invitrogen). RNA concentration and purity were assessed using a NanoDrop 2000 spectrophotometer (Thermo Fisher Scientific). For mRNA and lncRNA detection, reverse transcription was performed using the PrimeScript RT reagent Kit with gDNA Eraser (Takara, Shiga, Japan) to synthesize cDNA under the conditions of 37 °C for 15 min, 85 °C for 5 s. RT-qPCR was carried out using the SYBR Premix Ex Taq II Kit (Takara) on a StepOnePlus Real-Time PCR System. Reaction mixture (10 µL): 5 µL SYBR Premix Ex Taq II (2×), 0.4 µL ROX Reference Dye (50×), 0.4 µL forward and reverse primers (10 µM), 1 µL cDNA, 3.2 µL ddH₂O. PCR conditions: 30 s pre-denaturation at 95 °C; 5 s denaturation at 95 °C, 34 s annealing/extension at 60 °C, for a total of 40 cycles. The relative expression levels were calculated using the 2^−ΔΔCt^ method, with GAPDH as the internal control. For miRNA detection, reverse transcription was performed using the Mir-X miRNA First-Strand Synthesis Kit (Takara), and RT-qPCR was conducted using the Mir-X miRNA qRT-PCR SYBR Kit (Takara) with U6 as the internal control. Sequences for RT-qPCR (5’-3’) are as follows: LINC00578, forward: GGAGACTGCTAGAGGACCCA, and reverse: GGTAAAGGGTGGTGGAGGTG; miR-143-5p, forward: GCCGAGGGUGCAGUGCUGCA, and reverse: CTCAACTGGTGTCGTGGA; U6, forward: CTCGCTTCGGCAGCACA, and reverse: AACGCTTCACGAATTTGCGT; GAPDH, forward: GGCTGTTGTCATACTTCTCATGG, and reverse: GGAGCGAGATCCCTCCAAAAT.

### Cell culture and transfection

Human normal breast epithelial cell line MCF10A and breast cancer cell lines MCF-7, MDA-MB-231, and BT549 were obtained from ATCC (USA) and maintained in DMEM (Gibco) with 10% FBS (Gibco) at 37 °C under 5% CO₂. MCF10A cells require the addition of 10 µg/mL insulin (Sigma) and 20 ng/mL EGF (Sigma). SK‑BR‑3 cells were cultured in McCoy’s 5A medium (Gibco) supplemented with 10% FBS and 1% penicillin/streptomycin following ATCC official culture instructions. siRNAs targeting LINC00578 (si-LINC00578-1, si-LINC00578-2, si-LINC00578-3) and negative control siRNA (si-NC), as well as miR-143-5p mimic, miR-143-5p inhibitor, and their corresponding NCs were also purchased from GenePharma. Cells were seeded into 6-well plates (2 × 10^5^ cells/well) before transfection. When cell confluence reached 70–80%, transfection was performed. Transfections with 50 pmol of siRNA or 50 nM of miRNA mimic/inhibitor were performed using Lipofectamine 3000 (Invitrogen). Cells were harvested at 48 h post-transfection for subsequent experiments after verification of transfection efficiency using RT-qPCR.

### Dual-luciferase reporter assay

The wild-type (wt) and mutant (mut) LINC00578 sequences containing the predicted miR-143-5p binding site were synthesized and inserted into the pmirGLO vector (Promega, USA). MDA-MB-231 and BT549 cells were seeded in 24-well plates at a density of 5 × 10^4^ cells/well and co-transfected with the 200 ng reporter plasmids (wt-LINC00578 or mut-LINC00578) and 50 nM miR-143-5p mimic, 50 nM miR-143-5p inhibitor, or their respective NCs using Lipofectamine 3000. After 48 h, dual-luciferase activities were assessed with the Dual-Luciferase Reporter Assay System (Promega). Relative luciferase activity was calculated as the ratio of firefly luciferase signal to Renilla luciferase signal. Each group was set up with three biological replicates and three technical replicates.

### Cell proliferation assay

Cell proliferation was assessed with a Cell Counting Kit-8 (CCK-8, Dojindo, Japan). Transfected MDA-MB-231 and BT549 cells were seeded in 96-well plates at 2 × 10³ cells/well. After adding 10 µL of CCK-8 solution at 0, 24, 48, and 72 h, the cells were incubated for 2 h at 37 °C. Absorbance was then measured at 450 nm with a Bio-Rad microplate reader (Bio-Rad).

### Cell migration and invasion assays

Cell migration was evaluated using 8 μm transwell chambers (Corning, NY, USA) without Matrigel coating. Cell invasion was assessed using transwell chambers coated with Matrigel. Cells were digested with 0.25% trypsin (Gibco) 48 h after transfection, resuspended in serum-free DMEM, and adjusted to a density of 2 × 10⁵ cells/mL. Briefly, 200 µL of the transfected cell suspension was added to the upper chambers. Medium containing 10% FBS was added to the lower chambers as a chemoattractant. After incubation for 24 h, cells that migrated or invaded to the lower surface were fixed with methanol for 10 min, stained with 0.1% crystal violet for 15 min, and counted in five randomly selected fields of view under a microscope (Olympus, Tokyo, Japan).

### Statistical analysis

Statistical analyses were performed with SPSS 26.0 (IBM, USA) and GraphPad Prism 9.0 (GraphPad Software, USA). Categorical variables (e.g., genotype frequencies and clinical features) were compared using the chi-square test, while continuous data (such as gene expression, proliferation, migration, and invasion) were analyzed by Student’s t-test or one-way ANOVA. The diagnostic value of LINC00578 for breast cancer was evaluated using receiver operating characteristic (ROC) curve analysis. Associations between the LINC00578 rs7430456 polymorphism and breast cancer risk were quantified by odds ratios (ORs) and 95% confidence intervals (CIs) under co-dominant, dominant, recessive, and allelic genetic models. A *P*-value less than 0.05 was defined as statistically significant.

## Results

### Characteristics of study cohort

A total of 940 participants (480 cases and 460 controls) were included in this study. The cases and controls were well-matched on most baseline variables, including age and reproductive history (all *P* > 0.05). Notably, the groups diverged significantly in the prevalence of a positive family cancer history and the absence of breastfeeding, both of which were more common in the breast cancer group (*P* < 0.05 for both). The distribution of molecular subtypes within the patient cohort is detailed in Table 1. Among breast cancer patients, the molecular subtypes were distributed as 44.79% Luminal, 29.58% HER2-positive, and 25.63% TNBC.

**Table 1:** Basic parameters of participants

Parameters	Control (*n* = 460)	Breast cancer (*n* = 480)	*P* value
Age (years)	50.03 ± 9.49	51.01 ± 9.26	0.110
Age at menarche (years)	13.91 ± 1.56	14.00 ± 1.70	0.382
Age at menopause (years)	48.33 ± 3.70	48.99 ± 4.00	0.105
Menopause status			0.761
No	290 (63.04)	298 (62.08)	
Yes	170 (36.96)	182 (37.92)	
Number of pregnancies			0.693
< 2	185 (40.22)	187 (38.96)	
≥ 2	275 (59.78)	293 (61.05)	
Number of abortions			0.819
< 2	328 (71.30)	339 (70.62)	
≥ 2	132 (28.70)	141 (29.38)	
Breast-feeding history			0.032
No	83 (18.04)	114 (23.75)	
Yes	377 (81.96)	366 (76.25)	
Family history			0.038
No	438 (95.22)	441 (91.87)	
Yes	22 (4.78)	39 (8.13)	
Molecular subtype			
Luminal		215 (44.79)	
HER2-positive		142 (29.58)	
TNBC		123 (25.63)	

### Association between LINC00578 rs7430456 genotypes/alleles and breast cancer susceptibility

Table 2 presents the association of LINC00578 rs7430456 with breast cancer susceptibility, with the Hardy-Weinberg equilibrium (HWE) test confirming no deviation in the control group (*P* = 0.775). Under the co-dominant model, compared with the AA genotype (reference), the AG genotype was significantly associated with reduced breast cancer risk, and the GG genotype exhibited a stronger protective effect. In the dominant model (AG/GG vs. AA), the AG/GG genotype combination also correlated with lower breast cancer risk. Even in the recessive model (GG vs. AA/AG), the GG genotype remained associated with decreased breast cancer risk, though the effect was weaker. At the allelic level, the G allele was linked to a significantly reduced breast cancer risk compared with the A allele. Collectively, these results indicate that LINC00578 rs7430456 is associated with breast cancer susceptibility in the study cohort, with the G allele and GG/AG genotypes conferring a protective effect against breast cancer.

**Table 2:** Association between LINC00578 rs7430456 and breast cancer susceptibility

Genetic model	Control (*n* = 460)	Breast cancer (*n* = 480)	χ ^2^	OR (95%CI)	*P* value
AA	22 (4.78)	49 (10.21)	-	1	-
AG	162 (35.22)	175 (36.46)	0.485	0.281–0.838	0.009
GG	276 (60.00)	256 (53.33)	0.288	0.167–0.498	0.001
Dominant					
AA	22 (4.78)	49 (10.21)	-	1	-
AG/GG	438 (95.22)	431 (89.79)	0.442	0.263–0.743	0.002
Recessive					
AA/AG	184(40.00)	224(47.67)	-	1	-
GG	276 (60.00)	256 (53.33)	0.762	0.588–0.987	0.039
Alleles					
A	206 (22.39)	273 (28.44)	-	1	-
G	714 (77.61)	687 (71.56)	0.726	0.589–0.895	0.003
P^HWE^	0.775				

### LINC00578 expression patterns and diagnostic potential in breast cancer

**Fig. 1:**
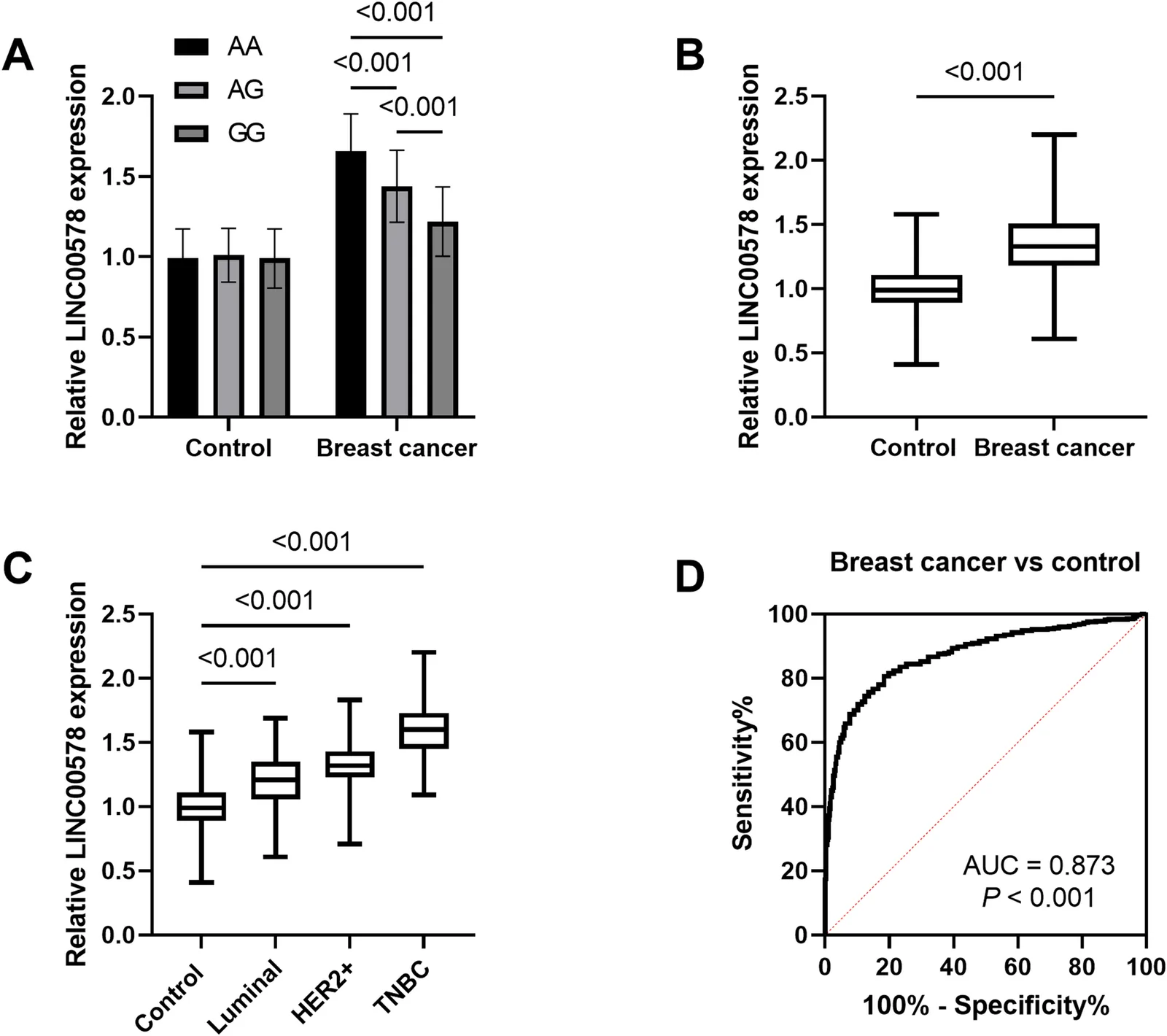
Expression profiles and diagnostic value of LINC00578 in breast cancer. **A **Breast cancer serum samples had higher LINC00578 expression in AA vs. AG/GG, with no difference in controls. **B** LINC00578 was upregulated in breast cancer (*n* = 480) vs. controls (*n* = 460, *P* < 0.001). **C** LINC00578 was upregulated in Luminal (*n* = 215), HER2+ (*n* = 142), TNBC (*n* = 123) vs. controls (all *P* < 0.001), highest in TNBC. **D** ROC curve for breast cancer diagnosis: AUC = 0.873 (95% CI: 0.845–0.901, *P* < 0.001). Data: mean ± SD. Stats: one-way ANOVA (**A, C**) or t-test (**B)**. All data were collected from the 480 breast cancer patients and 460 healthy Chinese Han volunteers enrolled in the present study

Figure 1 depicts the expression profiles and diagnostic utility of LINC00578 in breast cancer. The data in Fig. 1 were collected from our enrolled cohort consisting of 480 breast cancer patients and 460 healthy Chinese Han patients. LINC00578 rs7430456 genotype-dependent expression was displayed in Fig. 1A. As shown in Fig. 1A, among breast cancer patients, LINC00578 expression was significantly higher in the AA genotype group than in the AG and GG genotype groups, while no such difference was observed in healthy controls. In breast cancer, LINC00578 expression was highest in AA, intermediate in AG, and lowest in GG, concordant with the G allele’s protective role in breast cancer (Table 2). LINC00578 expression was significantly elevated in breast cancer versus controls (Fig. 1B). Figure 1C illustrates LINC00578 upregulation in Luminal, HER2-positive (HER2+), and TNBC subtypes compared to controls, with the most prominent increase in TNBC. The ROC curve for LINC00578 in breast cancer diagnosis, with an area under the curve (AUC) of 0.873 (Fig. 1D), signifying good diagnostic capability.

### Association between LINC00578 rs7430456 and molecular subtypes of breast cancer

Table 3 explores the association of LINC00578 rs7430456 with different molecular subtypes of breast cancer. The LINC00578 rs7430456 polymorphism was significantly associated with TNBC, HER2+, and Luminal breast cancer in terms of different genotypes, dominant model, and recessive model. In genotype analysis, compared with the AA genotype in the control group, the AG genotype and GG genotype in the TNBC group significantly reduced the risk of disease. Additionally, the GG genotype in the HER2 + group and Luminal group also showed significant associations. In the dominant model, the AG/GG genotypes in the TNBC group and Luminal group were significantly associated with the risk of disease. In the recessive model, only the GG genotype in the TNBC group exhibited a significant association. Allelic analysis revealed that the G allele was significantly associated with the reduced breast cancer risk in the TNBC group, while no significant associations were observed in the HER2 + group or Luminal group.

**Table 3:** The association between LINC00578 rs7430456 and molecular subtypes of breast cancer

SNP	TNBC (*n* = 123)	HER2+ (*n* = 142)	Luminal (*n* = 215)	Control (*n* = 460)	TNBC vs. control OR (95%CI); *P*	HER2 + vs. control OR (95%CI); *P*	Luminal vs. control OR (95%CI); *P*
Genotype							
AA	17 (13.82)	13 (9.15)	19 (8.84)	22 (4.78)	ref	ref	ref
AG	52 (42.28)	50 (35.21)	73 (33.95)	162 (35.22)	0.415 (0.205–0.841); 0.013	0.522(0.245,1.112); 0.088	0.522 (0.266–1.023); 0.056
GG	54 (43.90)	79 (55.63)	123 (57.21)	276 (60.00)	0.253 (0.126–0.508); <0.001	0.484(0.233–1.005); 0.048	0.516 (0.270–0.988); 0.043
Dominant							
AA	17 (13.82)	13 (9.15)	19 (8.84)	22 (4.78)	ref	ref	ref
AG/GG	106 (86.18)	129 (90.85)	196(91.16)	438 (95.22)	0.313 (00.161–0.611); <0.001	0.498 (0.244–1.017); 0.052	0.518 (0.274–0.979); 0.040
Recessive							
AA/AG	69 (56.10)	63 (44.37)	92 (42.79)	184 (40.00)	ref	ref	ref
GG	54 (43.90)	79 (55.63)	123 (57.21)	276 (60.00)	0.522 (0.349–0.780); 0.001	0.836(0.572–1.222); 0.355	0.891 (0.642–1.238); 0.492
Alleles							
A	86 (34.96)	76 (26.76)	111 (25.81)	206 (22.39)	ref	ref	ref
G	160 (65.04)	208 (73.24)	319 (74.19)	714 (77.61)	0.537 (0.396–0.728); <0.001	0.790(0.582–1.071); 0.129	0.829 (0.636–1.082); 0.167

### Association between LINC00578 expression and clinical characteristics of TNBC patients

Considering the significant correlation between LINC00578 expression and TNBC, the relationship between LINC00578 expression and clinical characteristics in 123 TNBC patients (grouped with median LINC00578 expression in TNBC patients) was further analyzed (Table 4). There were no significant associations between LINC00578 expression and age, menopause status, or tumor size. However, LINC00578 expression was associated with clinical stage, with a higher proportion of high LINC00578 expression in stage III-IV TNBC patients compared to stage I-II. Additionally, lymph node metastasis was also significantly correlated with LINC00578 expression, as TNBC patients with positive lymph node metastasis had a higher frequency of high LINC00578 expression than those with negative lymph node metastasis.

**Table 4:** Association between LINC00578 expression and clinical characteristics of TNBC patients

Characteristics	Cases	LINC00578 expression		*P*-value
	(*n* = 123)	Low (61)	High (62)	
Age (years)				0.789
< 50	59	30	29	
≥ 50	64	31	33	
Menopause				0.636
No	72	37	35	
Yes	51	24	27	
Tumor size (cm)				0.059
< 2	68	34	24	
≥ 2	65	27	38	
Clinical stage				0.011
I-II	77	45	32	
III-IV	46	16	30	
Lymph node metastasis				0.032
Negative	75	43	32	
Positive	48	18	30	

### Functional analysis of LINC00578 in TNBC cells

**Fig. 2:**
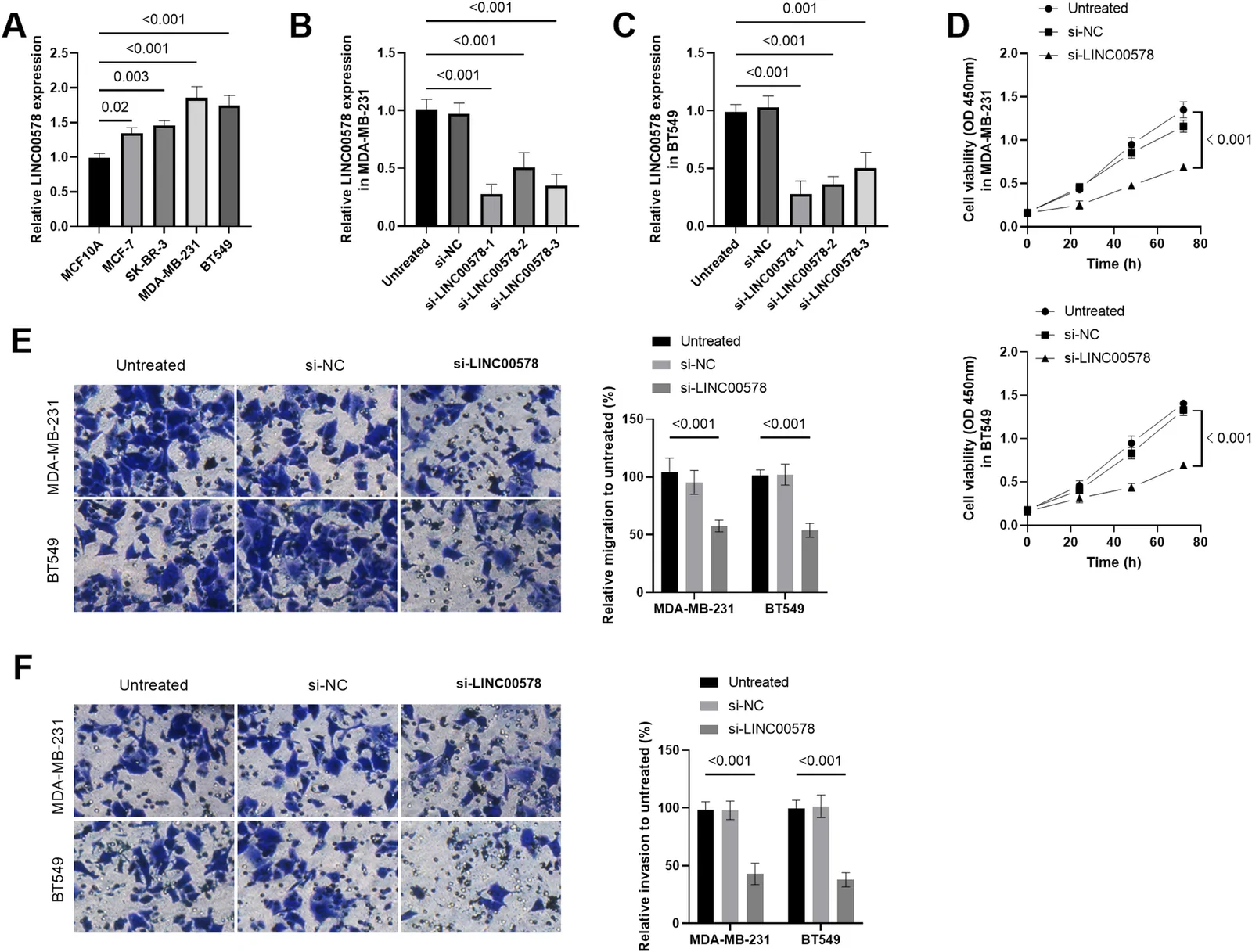
Functional role of LINC00578 in TNBC cells. **A **LINC00578 expression was higher in TNBC cells (MDA-MB-231, BT549) vs. normal (MCF10A) and non-TNBC cells. **B, C** si-LINC00578 reduced LINC00578 expression in MDA-MB-231/BT549. **D** LINC00578 knockdown inhibited TNBC cell proliferation. **E, F** LINC00578 knockdown suppressed TNBC cell migration and invasion (representative image 100 ×). Data: mean ± SD. Stats: one-way ANOVA. All experiments were performed with *n* = 3 independent biological replicates

Figure 2 investigates the functional role of LINC00578 in TNBC cells. Figure 2A shows that LINC00578 expression was significantly higher in TNBC cell lines (MDA-MB-231, BT549) than in normal breast epithelial cells (MCF10A) and non-TNBC cancer cell lines. Figures 2B and **C** demonstrate efficient knockdown of LINC00578 by siRNAs in MDA-MB-231 and BT549 cells, respectively. Among the three siRNAs, si-LINC00578-1 displayed the best transfection efficiency, which was chosen for the subsequent experiments. LINC00578 knockdown inhibited the proliferation of MDA-MB-231 and BT549 cells over time (Fig. 2D). LINC00578 knockdown suppressed migration and invasion of MDA-MB-231 and BT549 cells, respectively (Figs. 2E and **F**). Collectively, LINC00578 promotes TNBC cell proliferation, migration, and invasion.

### LINC00578 directly targets miR-143-5p in TNBC cells

**Fig. 3:**
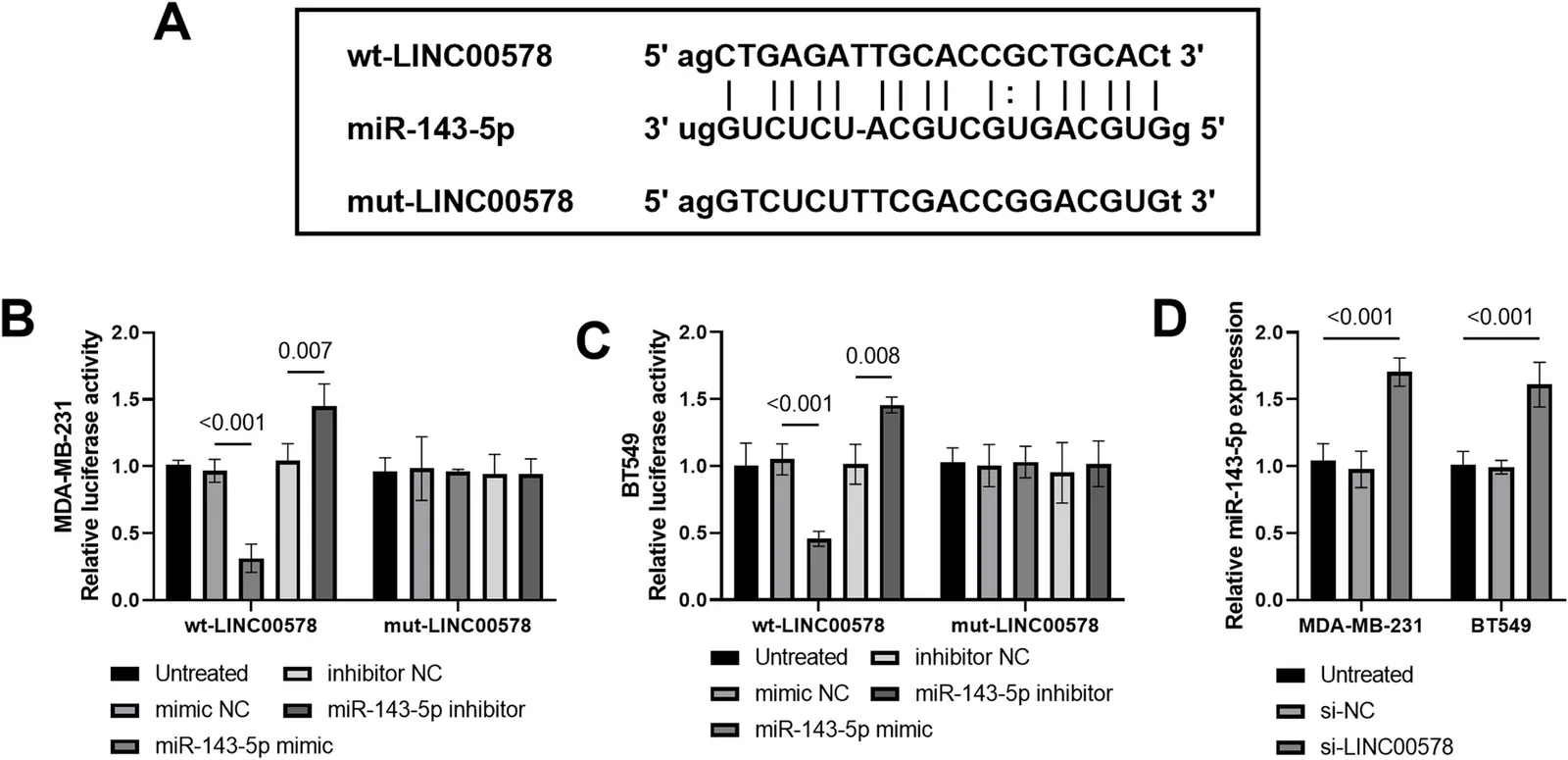
Interaction between LINC00578 and miR-143-5p in TNBC cells. **A **Predicted binding site of LINC00578 (wild-type/wt; mutant/mut) with miR-143-5p. **B, C** miR-143-5p mimic reduced wt-LINC00578 luciferase activity, no effect on mut-LINC00578 (MDA-MB-231/BT549). **D** LINC00578 knockdown upregulated miR-143-5p in TNBC cells. Data: mean ± SD. Stats: one-way ANOVA. All experiments were performed with *n* = 3 independent biological replicates

The direct interaction between LINC00578 and miR-143-5p in TNBC cells was investigated. Figure 3A presents the predicted binding site between wild-type LINC00578 (wt-LINC00578) and miR-143-5p, along with the mutated LINC00578 (mut-LINC00578) sequence used for validation. In cells transfected with wt-*LINC00578*, miR-143-5p mimic significantly reduced luciferase activity, while miR-143-5p inhibitor increased luciferase activity (Fig. 3B and C). No significant changes were observed in cells with mut-LINC00578 upon miR-143-5p mimic or inhibitor treatment, confirming specific binding. Figure 3D depicts miR-143-5p expression in MDA-MB-231 and BT549 cells after LINC00578 knockdown (si-LINC00578). Compared to the untreated and si-NC groups, miR-143-5p was significantly upregulated, indicating *LINC00578* sponges miR-143-5p to repress its expression.

### miR-143-5p mediates the oncogenic effects of LINC00578 in TNBC cells

**Fig. 4:**
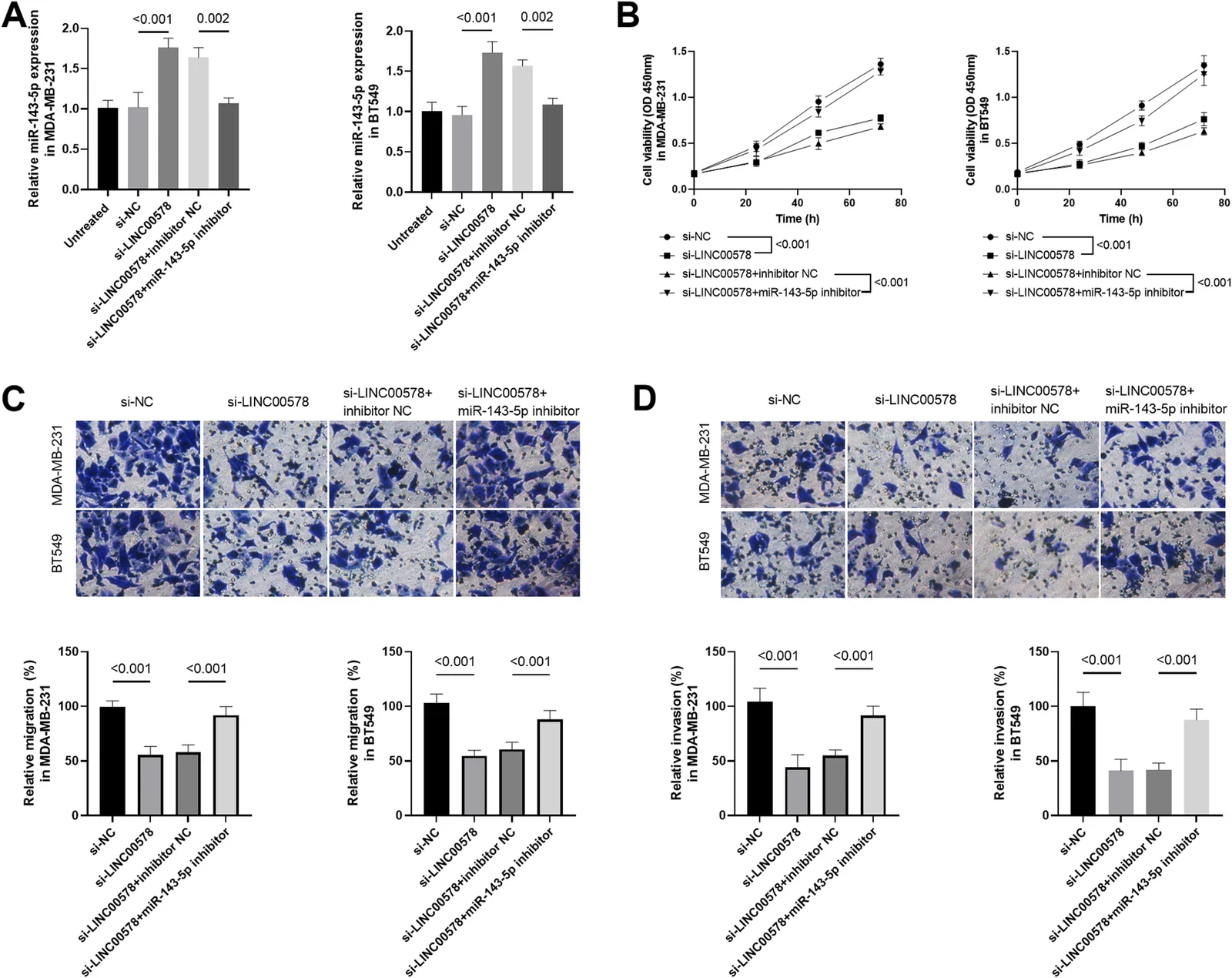
miR-143-5p mediates LINC00578’s oncogenic effects in TNBC cells. **A **LINC00578 knockdown upregulated miR-143-5p, reversed by miR-143-5p inhibitor. **B** LINC00578 knockdown inhibited proliferation, restored by the miR-143-5p inhibitor. **C, D** LINC00578 knockdown suppressed migration/invasion, reversed by miR-143-5p inhibitor (representative image 100 ×). Data: mean ± SD. Stats: one-way ANOVA. All experiments were performed with *n* = 3 independent biological replicates, and five random microscopic fields were counted for migration and invasion assays

LINC00578 knockdown significantly upregulated miR-143-5p, and this effect was reversed by miR-143-5p inhibitor (*P* = 0.002) (Fig. 4A). LINC00578 knockdown suppressed cell proliferation, while co-treatment with miR-143-5p inhibitor restored cell growth (Fig. 4B). LINC00578 knockdown inhibited cell migration and invasion, and these inhibitory effects were abrogated by miR-143-5p inhibitor (Fig. 4C and D). Collectively, these results indicate that LINC00578 exerts its oncogenic effects in TNBC cells by sponging miR-143-5p.

## Discussion

Breast cancer remains a leading cause of cancer-related mortality in women globally, characterized by marked molecular heterogeneity across subtypes, with TNBC posing a particular challenge due to the absence of targeted therapies [13]. lncRNAs have emerged as pivotal regulators in carcinogenesis, with accumulating evidence supporting their involvement in tumor initiation, progression, and therapeutic resistance [14, 15]. SNPs within lncRNA genomic regions can modulate lncRNA expression or functional interactions, thereby influencing cancer susceptibility and progression [16, 17]. In this study, LINC00578 rs7430456 and breast cancer risk, along with the functional role of LINC00578 in TNBC, were explored, offering novel insights into its potential as a biomarker and therapeutic target.

This study revealed that the LINC00578 rs7430456 G allele and AG/GG genotypes are linked with a reduced risk of breast cancer, which aligns with growing evidence that lncRNA-related SNPs can impact cancer susceptibility by altering lncRNA transcription, stability, or interactions with target molecules [6, 18]. For example, SNPs in lncRNA loci such as AQP4-AS1 and MALAT1 have been linked to altered expression patterns and increased risk of various cancers, including breast cancer [19, 20]. The protective effect of the G allele in rs7430456 may arise from its potential to regulate LINC00578 expression; further studies are required to confirm whether this SNP directly affects the transcriptional activity or splicing of LINC00578. Additionally, subgroup analyses indicated an association between rs7030456 and specific molecular subtypes, suggesting the polymorphism may exert subtype-specific effects, possibly due to differences in genetic backgrounds or microenvironment cues across subtypes [21]. The rs7430456 polymorphism is functionally relevant to breast cancer. It modulates the expression of LINC00578. The G allele inhibits LINC00578 expression, thereby suppressing the malignant phenotypes of TNBC cells mediated by the LINC00578/miR-143-5p axis, which accounts for its protective role against breast cancer. Although our data support a functional role for rs7430456 in regulating LINC00578 expression and breast cancer risk, it is important to consider the linkage disequilibrium (LD) context of this variant. Based on publicly available LD data from the 1000 Genomes Project Phase 3 (East Asian population), rs7430456 is not in strong LD (r² < 0.8) with other known regulatory variants in the LINC00578 locus. While we cannot formally exclude the possibility that the observed association is driven by another untyped variant in LD with rs7430456, our finding that the rs7430456 genotype correlates with LINC00578 expression in breast cancer patients supports the notion that rs7430456 may itself be a functional variant or a reliable proxy for the causal variant at this locus. Future targeted resequencing and fine-mapping studies will be needed to definitively identify the causal regulatory variant in this region.

This study detected significantly elevated levels of LINC00578 in the serum of breast cancer patients; however, the origin of this circulating protein warrants further investigation. The sources of lncRNA in serum typically involve three mechanisms: passive release following tumor cell necrosis or apoptosis; active secretion via microvesicles such as exosomes; and tumor-induced systemic changes. In pancreatic cancer research, the expression of LINC00578 in tumor tissue and serum showed a significant positive correlation, suggesting that serum levels can reflect its expression status in tumor tissue [22]. Furthermore, LINC00578 is significantly overexpressed in both breast cancer tissues and cell lines, and its expression is associated with clinical staging and metastasis in lung adenocarcinoma [23, 24]. In clear cell renal cell carcinoma, LINC00578 has been detected in urinary exosomes, suggesting that exosome-mediated active release may be a major route for its entry into the circulation (http://origin-gene.cn/database/exRNAdisease/detail.php?entry_ID=exR0410362). Based on the above evidence, we hypothesize that the elevated levels of LINC00578 in the serum of breast cancer patients primarily originate from tumor cells. This explanation provides a theoretical basis for the clinical application of LINC00578 as a circulating biomarker. LINC00578 upregulation in breast cancer serum samples (with the highest expression in TNBC) and its robust diagnostic efficacy support its utility as a breast cancer biomarker. This is consistent with reports of lncRNAs serving as robust diagnostic biomarkers in breast cancer. For instance, lncRNAs GAS5 and PVT1 are abnormally expressed in breast cancer and correlate with advanced disease stages [25, 26]. The upregulation of LINC00578 in TNBC, in particular, suggests it may play a unique role in the pathogenesis of this aggressive subtype, which is characterized by high genomic instability and limited treatment options. The high AUC value further supports LINC00578 as a promising diagnostic tool, potentially complementing existing markers like ER, PR, and HER2 to improve early detection, especially in TNBC, where early diagnosis is critical for survival.

Functional assays demonstrated that LINC00578 knockdown inhibits TNBC cell proliferation, migration, and invasion, indicating its oncogenic role in TNBC progression. This is in line with studies showing that lncRNAs promote cancer cell malignancy through diverse mechanisms [27, 28]. Mechanistically, we confirmed that LINC00578 directly interacts with miR-143-5p, a well-characterized tumor suppressor miRNA in breast cancer [11, 12]. MiR-143-5p functions in a tumor-suppressive role in inhibiting proliferation, invasion, and metastasis in gallbladder cancer and cervical cancer [29, 30]. This LINC00578/miR-143-5p axis adds to the growing ceRNA network in TNBC, explaining how LINC00578 overexpression derepresses oncogenic targets of miR-143-5p, driving malignant progression. Restoring miR-143-5p function, either directly or by inhibiting LINC00578, could thus represent a potential therapeutic strategy for TNBC.

This study has several limitations. First, the sample size was small, and our findings need validation in larger, multi-ethnic cohorts to confirm the generalizability of the rs7430456 association. Second, the oncogenic mechanism of LINC00578 in TNBC progression needs to be verified in in vivo studies. Third, this study did not directly measure the expression levels of LINC00578 in breast tumor tissue, nor did it isolate serum exosomes to verify the enrichment of LINC00578 in exosomes. Future studies should simultaneously analyze both tissue and serum samples and conduct exosome isolation experiments to determine the circulating carrier form of LINC00578. Finally, the functional impact of rs7430456 on LINC00578 expression or structure warrants investigation, as this could clarify the link between the polymorphism and cancer risk.

## Conclusions

In summary, this study identifies LINC00578 rs7430456 as a potential genetic marker for breast cancer susceptibility and demonstrates that LINC00578 promotes TNBC progression by sponging miR-143-5p. These findings highlight LINC00578 as a promising diagnostic biomarker and therapeutic target, particularly for TNBC, and provide a foundation for future studies exploring its clinical utility.

## Data Availability

All data generated or analyzed during this study are included in this article. Further enquiries can be directed to the corresponding author.
